# Preprocedural D-Dimer Level as a Predictor of First-Pass Recanalization and Functional Outcome in Endovascular Treatment of Acute Ischemic Stroke

**DOI:** 10.3390/jcm12196289

**Published:** 2023-09-29

**Authors:** Jang-Hyun Baek, Ji Hoe Heo, Hyo Suk Nam, Byung Moon Kim, Dong Joon Kim, Young Dae Kim

**Affiliations:** 1 Department of Neurology, Kangbuk Samsung Hospital, Sungkyunkwan University School of Medicine, Seoul 03181, Republic of Korea; janghyun.baek@gmail.com; 2 Department of Neurology, Severance Stroke Center, Severance Hospital, Yonsei University College of Medicine, Seoul 03722, Republic of Korea; jhheo@yuhs.ac (J.H.H.); hsnam@yuhs.ac (H.S.N.); 3 Interventional Neuroradiology, Department of Radiology, Severance Stroke Center, Severance Hospital, Yonsei University College of Medicine, Seoul 03722, Republic of Korea; bmoon21@hanmail.net (B.M.K.); djkimmd@yuhs.ac (D.J.K.)

**Keywords:** D-dimer, endovascular treatment, thrombectomy, stroke, outcome

## Abstract

We aimed to evaluate the association between preprocedural D-dimer levels and endovascular and clinical outcomes. We retrospectively reviewed patients with acute intracranial large-vessel occlusion who underwent mechanical thrombectomy. Plasma D-dimer levels were measured immediately before the endovascular procedure. Endovascular outcomes included successful recanalization, first-pass recanalization (first-pass effect (FPE) and modified FPE (mFPE)), thrombus fragmentation, and the number of passes of the thrombectomy device. Clinical outcomes were assessed at 3 months using the modified Rankin Scale. A total of 215 patients were included. Preprocedural D-dimer levels were lower in patients with FPE (606.0 ng/mL [interquartile range, 268.0–1062.0]) than in those without (879.0 ng/mL [437.0–2748.0]; *p* = 0.002). Preprocedural D-dimer level was the only factor affecting FPE (odds ratio, 0.92 [95% confidence interval, 0.85–0.98] per 500 ng/mL; *p* = 0.022). D-dimer levels did not differ significantly based on successful recanalization and thrombus fragmentation. The number of passes of the thrombectomy device was higher (*p* = 0.002 for trend) and the puncture-to-recanalization time was longer (*p* = 0.044 for trend) as the D-dimer levels increased. Patients with favorable outcome had significantly lower D-dimer levels (495.0 ng/mL [290.0–856.0]) than those without (1189.0 ng/mL [526.0–3208.0]; *p* < 0.001). Preprocedural D-dimer level was an independent factor for favorable outcome (adjusted odds ratio, 0.88 [0.81–0.97] per 500 ng/mL; *p* = 0.008). In conclusion, higher preprocedural D-dimer levels were significantly associated with poor endovascular and unfavorable functional outcomes.

## 1. Introduction

Plasma D-dimer is a fibrin degradation product derived from plasmin-mediated fibrinolysis [1]. D-dimer levels increase in thrombogenic conditions, such as deep vein thrombosis, pulmonary embolism, and active cancer [2,3,4,5]. For acute ischemic stroke, elevated D-dimer levels are significantly associated with poor prognosis (such as stroke progression, lesion enlargement, and unfavorable functional outcome) and specific stroke etiologies (such as cancer-related stroke) [6,7,8,9,10].

Mechanical thrombectomy primarily targets a thrombus in the occluded artery; thus, the success of endovascular treatment may depend on the characteristics of the thrombus. Because plasma D-dimer reflects the activation of the coagulation pathway and corresponding hyperfibrinolysis, the conditions of thrombus formation might be altered by different D-dimer levels. For example, in cancer-related stroke, which always accompanies a fairly high D-dimer level, platelet-rich or white thrombi are predominantly observed [11,12]. Presumably, plasma D-dimer levels might affect endovascular and further clinical outcomes through various thrombogenic conditions or types of thrombus. However, the clinical significance of preprocedural D-dimer levels remains uncertain in endovascular treatment, especially for mechanical thrombectomy [13].

Accordingly, we hypothesized that endovascular and clinical outcomes would be affected by preprocedural D-dimer levels. We aimed to evaluate the associations between preprocedural D-dimer levels and (1) various endovascular outcomes beyond successful recanalization and (2) functional outcome.

## 2. Materials and Methods

### 2.1. Study Population

We retrospectively reviewed consecutive patients with acute stroke and intracranial occlusion who underwent endovascular treatment between 2019 and 2021. Patients were selected from a prospectively maintained stroke registry at a tertiary stroke center. Endovascular treatment was generally considered for patients who met the following criteria: (1) a computed tomography (CT)-angiography-determined endovascularly accessible intracranial vessel occlusion associated with neurological symptoms; (2) age ≥ 19 years; (3) baseline National Institutes of Health Stroke Scale (NIHSS) score ≥ 4; (4) time from stroke onset to groin puncture < 24 h; (5) preprocedural CT-Alberta Stroke Program Early Computed Tomography Score (CT-ASPECTS) ≥ 6; (6) patients with time from stroke onset > 6 h. The eligibility criteria of the DWI or CTP Assessment with Clinical Mismatch in the Triage of Wake-Up and Late Presenting Strokes Undergoing Neurointervention with Trevo (DAWN) and Diffusion and Perfusion Imaging Evaluation for Understanding Stroke Evolution (DEFUSE 3) trials were also considered. We preferably performed endovascular treatment in patients with premorbid modified Rankin Scale (mRS) scores ≤ 3. Patients eligible for intravenous tissue-type plasminogen activator (tPA) treatment were treated with 0.9 mg/kg tPA.

For this study, we included patients who (1) had a proximal vessel occlusion, including the intracranial internal carotid artery, middle cerebral artery, vertebral artery, and basilar artery; (2) were treated with mechanical thrombectomy; and (3) had a preprocedural D-dimer level before endovascular treatment.

### 2.2. Mechanical Thrombectomy Procedure

All endovascular procedures were performed under local anesthesia, and conscious sedation was administered when necessary. Mechanical thrombectomy was performed according to previous recommendations [14,15,16]. An 8- or 9-F balloon guide catheter (BGC) was routinely used. A distal access catheter was occasionally used when necessary. The choice between stent retriever and contact aspiration thrombectomy was made at the discretion of the operator.

For stent retriever thrombectomy, several types of stent retrievers—Solitaire^®^ (Medtronic, Minneapolis, MN, USA), Trevo^®^ (Stryker, Kalamazoo, MI, USA), EmboTrap^®^ (Cerenovus, Irvine, CA, USA), Aperio^®^ (Acandis, Pforzheim, Germany), and Revive^®^ (Codman Neuro/DePuy Synthes, Raynham, MA, USA)—were used. The stent retriever was delivered and deployed over the thrombus using a 0.021- or 0.027-inch microcatheter. The stent retriever was deployed for a few minutes before retrieval. For retrieval, the balloon of the BGC was inflated, and the stent retriever and microcatheter were cautiously retrieved under constant aspiration through the BGC using a 20 or 50 mL syringe. For contact aspiration thrombectomy, a few types of aspiration catheters—Penumbra^®^ (Penumbra, Alameda, CA, USA), Catalyst^®^ 6 (Stryker, Kalamazoo, MI, USA), and Sofia^®^ (Microvention, Aliso Viejo, CA, USA)—were used. The aspiration catheter was advanced as close as possible to the proximal end of the thrombus using a coaxial technique with a microcatheter and a microwire. Contact aspiration was then performed manually using a 50 mL syringe. Concurrent contact aspiration with stent retriever thrombectomy (e.g., Solumbra, ARTS, and SAVE techniques) was allowed but was rarely performed, and only for intractable cases. These processes were repeated until a modified Thrombolysis In Cerebral Infarction (mTICI) grade of 2b or 3 was achieved. The time to discontinue the attempts or switch to another endovascular modality was determined by the operator’s judgment, considering the occlusion pathogenesis and clinical or patient condition, among other factors.

### 2.3. Study Variables and Outcomes

Plasma D-dimer levels (ng/mL) were immediately measured as part of a routine laboratory test in the emergency room upon admission. Approximately 4 mL of venous blood was collected in a sample tube, anticoagulated using sodium citrate, and centrifuged at 3000 rpm for 10 min to isolate the serum. D-dimer levels were measured using an immunoturbidimetric assay with an automatic biochemical detector.

For endovascular outcomes, we assessed the following endovascular findings: successful recanalization, first-pass recanalization (first-pass effect (FPE) and modified first-pass effect (mFPE)), thrombus fragmentation, and number of passes of the thrombectomy device. Successful recanalization was defined as a final mTICI grade of 2b or 3, without further reocclusion during the procedure. FPE was defined as near-complete or complete revascularization (mTICI grade 2c or 3) after the first pass of the thrombectomy device [17,18]. For FPE, first-pass mTICI grades 2c or 3 should be maintained without additional treatment; mFPE was defined in a less restrictive manner, as mTICI grade 2b, 2c, or 3 after the first pass of the thrombectomy device. Thrombus fragmentation by the mechanical thrombectomy procedure was determined by angiographic findings. Angiographies were assessed immediately after each thrombectomy attempt. Any downstream occlusions newly observed after passing the thrombectomy device were defined as thrombus fragmentation [19,20]. Multiple intracranial occlusions before starting the endovascular procedure were also considered evidence of thrombus fragmentation. Two independent neurointerventionalists, who were blinded to the clinical information and follow-up imaging, assessed the recanalization results and thrombus fragmentation. The κ-values for the inter-rater reliability were calculated. All discrepancies were resolved by consensus.

Functional outcome was assessed based on the mRS score at 3 months after stroke onset. Favorable outcome was defined as mRS scores of 0, 1, or 2. The mRS scores were primarily evaluated by stroke neurologists during the patient’s routine clinical follow-up at 3 months (±2 weeks). If a patient could not visit the clinic, a stroke neurologist or trained nurse interviewed the patient or their family via telephone to determine the mRS score.

### 2.4. Statistical Analysis

First, to evaluate the association between preprocedural D-dimer levels and endovascular outcomes, D-dimer levels were compared based on successful recanalization, FPE, mFPE, and thrombus fragmentation. We also performed trend analysis to observe changes in clinical and endovascular findings according to D-dimer levels. D-dimer levels were categorized into three groups according to the tertile values. For endovascular outcomes showing significance, multivariable logistic regression analysis was additionally performed to observe the independence of preprocedural D-dimer levels for significant endovascular outcomes, by adjusting for other variables with *p* < 0.1 in the univariable analyses. To determine the predictive power of preprocedural D-dimer levels for significant endovascular outcomes, we performed receiver operating characteristic (ROC) curve analysis and calculated the area under the ROC curve (AUC). The optimal cutoff D-dimer level for a significant endovascular outcome was determined using the Youden index. Second, a similar analysis was performed to determine the association between preprocedural D-dimer levels and functional outcome.

Statistical significance was set at *p* < 0.05, with a 95% confidence interval (CI). All statistical analyses were performed using R software (version 4.2.3; R Foundation, https://www.r-project.org, accessed on 23 August 2023).

## 3. Results

**Figure 1:**
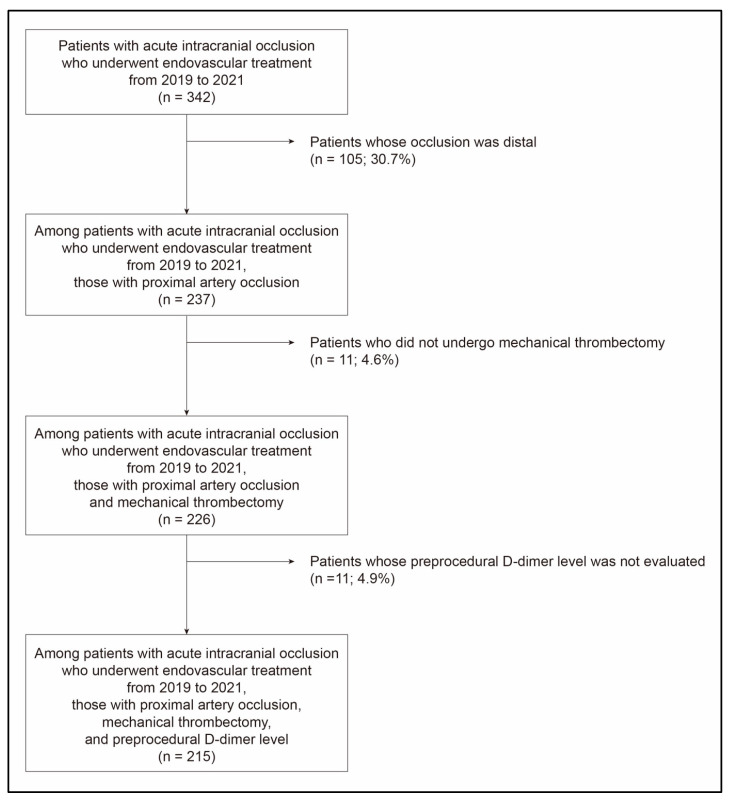
Patient selection flowchart.

A total of 215 patients (mean age, 71.3 ± 12.9 years; male, 52.6%) were ultimately included (Figure 1). Their initial NIHSS score was 14.0 [interquartile range (IQR), 9.0–19.0] (Table 1). The most common location of occlusion was the middle cerebral artery (54.4%). Twenty-six patients (12.1%) had posterior circulation stroke. Intravenous tPA was administered to 68 patients (31.6%). The median time from onset to groin puncture was 352.0 min [IQR, 170.0–719.0]. BGC was used in 183 patients (85.1%). The κ-values for the inter-rater reliability were 0.91, approximately 0.82, and 0.79 for FPE, mFPE and successful recanalization, and thrombus fragmentation, respectively. Successful recanalization was achieved in 203 patients (94.4%), and FPE was achieved in 71 patients (33.0%). Eighty-seven patients had favorable outcome (40.5%). The median preprocedural D-dimer level in the study population was 757.0 ng/mL [IQR, 392.0–2562.5].

### 3.1. Preprocedural D-dimer Levels and Endovascular Outcomes

**Figure 2:**
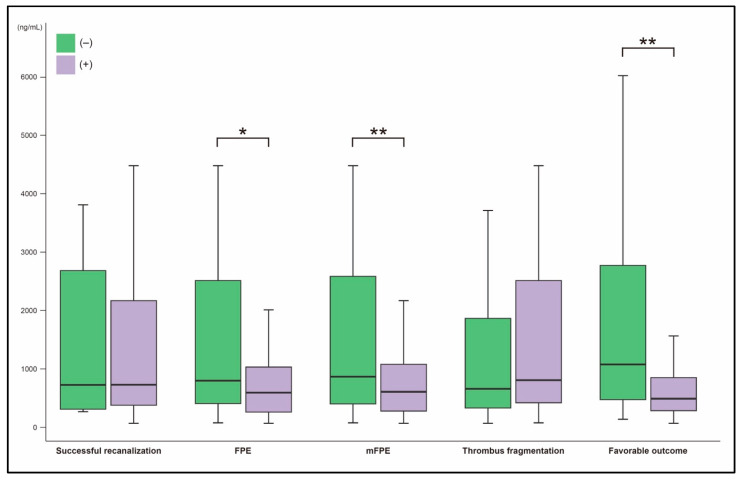
Comparison of preprocedural D-dimer levels according to endovascular and clinical outcomes; * *p*-value < 0.01; ** *p*-value < 0.001. FPE, first-pass effect; mFPE, modified first-pass effect.

The preprocedural D-dimer levels were significantly different between patients with and without FPE. Patients with FPE had lower D-dimer levels (606.0 ng/mL [IQR, 268.0–1062.0] vs. 879.0 ng/mL [437.0–2748.0]; *p* = 0.002; Figure 2). D-dimer levels were also lower in patients with mFPE (614.0 [292.0–1138.0] vs. 1126.0 [417.0–2958.0]; *p* = 0.001). D-dimer levels did not differ based on successful recanalization (757.0 [392.0–2520.0] in patients with successful recanalization vs. 732.0 [315.0–2690.0] in those without successful recanalization; *p* = 0.962) or thrombus fragmentation (883.0 [436.0–2716.0] in patients with thrombus fragmentation vs. 712.0 [373.0–2237.0] in patients without thrombus fragmentation; *p* = 0.129; Figure 2).

In the trend analysis, FPE and mFPE were achieved significantly less as the D-dimer levels increased; the frequency of FPE decreased from 41.7% to 21.1% (*p* = 0.009; Table 2), while that of mFPE decreased from 48.6% to 28.2% (*p* = 0.014). The number of passes of the thrombectomy device was higher in the groups with higher D-dimer levels (1.8 ± 1.4, 2.0 ± 1.4, and 2.7 ± 1.5 for each tertile group, respectively; *p* = 0.002). The time from groin puncture to recanalization was longer in the groups with higher D-dimer levels (28.0 [18.0–48.0], 30.0 [24.0–54.0], and 37.5 [23.2–61.2] for each tertile group, respectively; *p* = 0.044). Successful recanalization and thrombus fragmentation did not significantly change with D-dimer levels.

**Figure 3:**
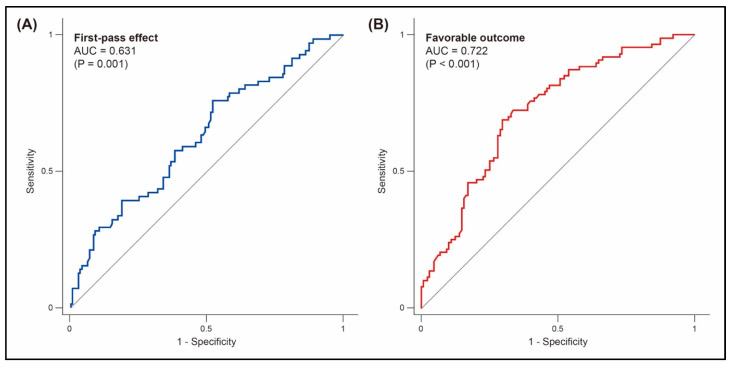
Prediction of first-pass effect (**A**) and favorable outcome (**B**) by preprocedural D-dimer levels. AUC, area under the receiver operating characteristic curve.

Preprocedural D-dimer level was the only clinical factor affecting FPE (odds ratio, 0.92 [95% CI, 0.85–0.98] per 500 ng/mL; *p* = 0.022; Table 3). No other clinical factors were significantly different between patients with and without FPE. Preprocedural D-dimer levels could predict FPE (AUC value, 0.631; cutoff value, 1085.0 ng/mL; sensitivity, 76.1%; specificity, 47.9%; *p* = 0.001; Figure 3).

### 3.2. Preprocedural D-dimer Levels and Functional Outcome

Patients with favorable outcome had significantly lower D-dimer levels than those without (495.0 [290.0–856.0] vs. 1189.0 [526.0–3208.0]; *p* < 0.001; Figure 2 and Table 4). As D-dimer levels increased, functional outcome tended to be less favorable. Patients with favorable outcome accounted for 58.3%, 45.8%, and 16.9% in each tertile group, respectively (*p* < 0.001; Table 2). In the multivariable analysis, preprocedural D-dimer level was an independent factor for favorable outcome (adjusted odds ratio, 0.88 [95% CI, 0.81–0.97] per 500 ng/mL; *p* = 0.008; Table 5). Preprocedural D-dimer levels could predict favorable outcome (AUC, 0.722; cutoff value, 667.0 ng/mL; sensitivity, 67.0%; specificity, 70.3%; *p* < 0.001; Figure 3).

## 4. Discussion

This study comprehensively evaluated endovascular outcomes associated with preprocedural D-dimer levels. We found that preprocedural D-dimer levels were significantly associated with endovascular and functional outcomes after mechanical thrombectomy. Higher preprocedural D-dimer levels appeared to have adverse effects on endovascular outcomes. First-pass recanalization, including FPE and mFPE, was less likely to be achieved in patients with high D-dimer levels. Moreover, they required more passes of the thrombectomy devices and took a longer time to achieve successful recanalization. Functional outcome also became less favorable as D-dimer levels increased. Preprocedural D-dimer level may be an ancillary marker for predicting endovascular and clinical outcomes after mechanical thrombectomy.

The histological features of the thrombus may be a reason for poor endovascular outcomes. *First*, higher D-dimer levels indicate an active thrombogenic condition mediated by fibrin. Particularly in cancer-related stroke, D-dimer levels are markedly elevated and are a relevant marker for stroke prognosis or pathomechanism [10,21,22,23]. Thrombi in cancer-related stroke have been known to be platelet- or fibrin-rich [12,24]. Fibrin-rich thrombi are more resistant to thrombectomy procedures than erythrocyte-rich thrombi [25,26,27,28,29,30]. More simply, a larger thrombus can be formed under active thrombogenic conditions with a higher D-dimer level. *Second*, in this study, patients with higher D-dimer levels required more passes of the thrombectomy devices, *even without* thrombus fragmentation or subsequent downstream occlusions. This suggests that poor endovascular outcomes in patients with higher D-dimer levels might result from an intractable response to primary occlusion. Poor engagement of thrombectomy devices with the thrombus might be a crucial background for intractability, which is a feature of a firm or fibrin-rich thrombus.

The relationship between preprocedural D-dimer levels and successful recanalization has been inconsistent in previous studies. Preprocedural D-dimer levels did not differ significantly depending on successful recanalization [13,21,22]. Successful recanalization was conditionally more frequent only in patients with D-dimer levels above the threshold value of successful recanalization [13,23]. In this study, successful recanalization was not associated with preprocedural D-dimer levels; moreover, these levels did not significantly predict successful recanalization and showed unacceptable predictability. Regardless of first-pass recanalization failure, final successful recanalization could be achieved by further endovascular procedures, even at higher D-dimer levels. In fact, among 144 patients who did not achieve FPE, 132 (91.7%) were able to achieve final successful recanalization through further thrombectomy attempts. It might be possible that plasma D-dimer levels can be a significant but relatively weak determinant of poor endovascular responses. First-pass recanalization might be a more sensitive marker representing endovascular easiness.

Although a few reports have already evaluated endovascular outcomes in association with plasma D-dimer levels, this study still seems valuable. Recently, more rigorous endovascular outcomes have been highlighted for the best endovascular performance. Considering that first-pass recanalization is widely regarded as a technical goal in current practice, this study could give helpful insights for better endovascular performance.

Poor endovascular outcomes due to high D-dimer levels can be the most relevant mediator of unfavorable functional outcome. The achievement of FPE is crucial for favorable outcome, even after adjusting for other clinical and endovascular variables. Considering that preprocedural D-dimer level was the only factor affecting FPE in this study, the influence of preprocedural D-dimer level on functional outcome through FPE seems plausible.

However, preprocedural D-dimer level was also an independent factor for functional outcome, irrespective of FPE. This suggests that it may affect the functional outcome in another way, but not through FPE. *First*, D-dimer level is a well-known prognostic factor in common acute ischemic stroke. Higher D-dimer levels are adversely associated with the progression of lesion size, functional disability, mortality, and stroke recurrence [7,31,32,33,34,35]. *Second*, higher D-dimer levels are also associated with poor clinical outcomes *after* mechanical thrombectomy [5,36]. Elevated D-dimer levels reflect hypercoagulable status and subsequent active fibrinolysis, which, in turn, may play a significant role in hemorrhagic transformation after mechanical thrombectomy. In fact, the D-dimer levels were found to be higher in patients with symptomatic intracerebral hemorrhage and were predictive of hemorrhagic transformation [5]. Simply, preprocedural D-dimer levels could predict functional outcome after mechanical thrombectomy. Interestingly, the reported predictability was similar to that observed in our study [36]. *Third*, the prognostic value of preprocedural D-dimer levels has also been evaluated actively in cancer-related stroke. Higher D-dimer levels are consistently associated with unfavorable functional outcome after mechanical thrombectomy in patients with cancer-related stroke [21,22,23]. Because elevated D-dimer levels are a hallmark of cancer-related stroke, patients with higher D-dimer levels might have an underlying or hidden malignancy, which could have contributed to the unfavorable functional outcome in our study.

This study had some limitations. *First*, as this study was performed retrospectively, the endovascular procedures were not protocolized, and many were decided at the operator’s discretion. This may have affected the endovascular outcomes in this study. However, the endovascular procedures were performed homogeneously in a single center. We strictly followed the general common methods for mechanical thrombectomy and attempted to minimize variations, such as combining a stent retriever and an aspiration catheter. Moreover, stent retriever thrombectomy was chosen as the frontline treatment technique in most cases, and Solitaire^®^ was exclusively used. Thus, FPE, the principal endovascular outcome in this study, did not appear to be seriously affected by the retrospective study design.

*Second*, although the occlusion pathomechanism or etiology can affect endovascular outcomes, these were not controlled. First-pass recanalization is relatively uncommon in patients with intracranial atherosclerosis (ICAS)-related occlusion, because stent retriever thrombectomy is not as effective as embolic occlusion [37]. However, the relationship between ICAS-related occlusion, first-pass recanalization, and D-dimer levels is not simple, because D-dimer levels are known to be lower in ICAS-related occlusions [38]. In spite of this inverse relationship, lower preprocedural D-dimer levels were associated with better first-pass recanalization in this study.

*Third*, although some clinical conditions, such as deep vein thrombosis, chronic kidney disease, and recent cardiac/pulmonary decompensation or surgery, can highly affect plasma D-dimer levels, we did not exclude them in this study. However, we originally tried to evaluate the significance of preprocedural D-dimer levels irrespective of such clinical conditions. Moreover, even after excluding such patients, the significance of preprocedural D-dimer levels was not changed.

*Fourth*, as mentioned earlier, this study did not examine the thrombus histology. The histological features of a thrombus can be a clue to understanding poor endovascular outcomes due to higher D-dimer levels. Mechanical thrombectomy clearly depends on the physical properties of the thrombus, which are determined by its histological composition [25,26]. If plasma D-dimer is involved in a particular step of the coagulation pathway or affects the environment of thrombus formation, it could be a surrogate marker representing specific histological features of the thrombus. Because plasma D-dimer is a common finding in acute ischemic stroke, it would be useful to predict the procedural response to mechanical thrombectomy maneuvers and subsequent endovascular outcomes. However, no study has yet assessed the relationship between D-dimer levels and thrombus histology. Further studies are necessary to evaluate the role of plasma D-dimer levels in determining thrombus composition and physical properties.

## 5. Conclusions

Higher preprocedural D-dimer levels were significantly associated with poor endovascular outcomes, including less first-pass recanalization (FPE and mFPE), more passes of the thrombectomy device, and a longer time from groin puncture to recanalization. They were also an independent risk factor for unfavorable functional outcome. Therefore, preprocedural D-dimer level may be a prognostic marker for endovascular and clinical outcomes after mechanical thrombectomy.

## Figures and Tables

**Table 1:** Characteristics of the study population.

	*n* = 215
**Demographics and stroke risk factors**	
Age (years)	71.3 (±12.9)
Men	113 (52.6)
Hypertension	158 (73.5)
Diabetes	71 (33.0)
Dyslipidemia	54 (25.1)
Current smoking	31 (14.4)
Coronary artery occlusive disease	30 (14.0)
Atrial fibrillation	112 (52.1)
**Clinical conditions**	
Initial NIHSS score	14.0 [9.0; 19.0]
Intravenous tPA administration	68 (31.6)
Location of occlusions	
Internal carotid artery	72 (33.5)
Middle cerebral artery	117 (54.4)
Vertebral artery	3 (1.4)
Basilar artery	23 (10.7)
Onset-to-puncture time (minutes)	352.0 [170.0; 719.0]
Use of balloon guide catheter	183 (85.1)
**Endovascular outcomes**	
Successful recanalization	203 (94.4)
mTICI grades	
0	5 (2.3)
1	1 (0.5)
2a	6 (2.8)
2b	65 (30.2)
3	138 (64.2)
Puncture-to-recanalization time (minutes)	31.0 [20.0; 54.0]
First-pass recanalization	
Modified first-pass effect	90 (41.9)
First-pass effect	71 (33.0)
Thrombus fragmentation	79 (36.7)
Number of passes of thrombectomy device	2.2 (±1.5)
**Favorable outcome**	87 (40.5)

Values represent the mean and standard deviation (±) or number of patients (in %). Values in brackets represent the first and third quartiles for median values. NIHSS, National Institutes of Health Stroke Scale; tPA, tissue-type plasminogen activator; mTICI, modified Thrombolysis In Cerebral Infarction.

**Table 2:** Comparison of clinical and endovascular findings according to preprocedural D-dimer levels.

	Tertile 1 284.0 ng/mL [216.5; 392.0] (*n* = 72)	Tertile 2 761.5 ng/mL [615.2; 1022.2] (*n* = 72)	Tertile 3 3285.0 ng/mL [2594.0; 7206.0] (*n* = 71)	*p*-Value *
**Demographics and stroke risk factors**				
Age (years)	68.0 (±13.8)	73.7 (±11.8)	72.0 (±12.4)	0.058
Men	46 (63.9)	39 (54.2)	28 (39.4)	0.004
Hypertension	52 (72.2)	52 (72.2)	54 (76.1)	0.605
Diabetes	23 (31.9)	22 (30.6)	26 (36.6)	0.555
Dyslipidemia	24 (33.3)	17 (23.6)	13 (18.3)	0.039
Current smoking	14 (19.4)	10 (13.9)	7 (9.9)	0.103
Coronary artery occlusive disease	9 (12.5)	14 (19.4)	7 (9.9)	0.654
Atrial fibrillation	29 (40.3)	45 (62.5)	38 (53.5)	0.112
**Clinical conditions**				
Initial NIHSS score	10.5 [6.0; 16.0]	15.0 [9.8; 19.2]	15.0 [12.0; 19.5]	<0.001
Intravenous tPA administration	10 (13.9)	31 (43.1)	27 (38.0)	0.002
Location of occlusions				0.589
Internal carotid artery	18 (25.0)	30 (41.7)	24 (33.8)	
Middle cerebral artery	44 (61.1)	35 (48.6)	38 (53.5)	
Vertebral artery	9 (12.5)	6 (8.3)	8 (11.3)	
Basilar artery	1 (1.4)	1 (1.4)	1 (1.4)	
Onset-to-puncture time (minutes)	531.0 [266.0; 908.0]	308.0 [166.0; 657.0]	265.0 [160.0; 650.0]	0.007
Use of balloon guide catheter	59 (81.9)	64 (88.9)	60 (84.5)	0.664
**Endovascular outcomes**				
Successful recanalization	67 (93.1)	70 (97.2)	66 (93.0)	0.984
Puncture-to-recanalization time (minutes)	28.0 [18.0; 48.0]	30.0 [24.0; 54.0]	37.5 [23.2; 61.2]	0.044
Complete recanalization	46 (63.9)	51 (70.8)	41 (57.7)	0.448
First-pass recanalization				
Modified first-pass effect	35 (48.6)	35 (48.6)	20 (28.2)	0.014
First-pass effect	30 (41.7)	26 (36.1)	15 (21.1)	0.009
Thrombus fragmentation	23 (31.9)	24 (33.3)	32 (45.1)	0.105
Number of passes of thrombectomy device	1.8 (±1.4)	2.0 (±1.4)	2.7 (±1.5)	0.002
**Favorable outcome**	42 (58.3)	33 (45.8)	12 (16.9)	<0.001

Values represent the mean and standard deviation (±), median with the first and third quartiles [in brackets], or the number of patients (in %); * *p*-value for trend. NIHSS, National Institutes of Health Stroke Scale; tPA, tissue-type plasminogen activator.

**Table 3:** Comparison of clinical and endovascular findings according to the first-pass effect.

	First-Pass Effect (−) (*n* = 144)	First-Pass Effect (+) (*n* = 71)	*p*-Value
**Factors that might affect first-pass effect**			
**Demographics and stroke risk factors**			
Age (years)	70.9 (±12.3)	72.0 (±13.9)	0.551
Men	75 (52.1)	38 (53.5)	0.843
Hypertension	104 (72.2)	54 (76.1)	0.549
Diabetes	43 (29.9)	28 (39.4)	0.160
Dyslipidemia	31 (21.5)	23 (32.4)	0.084
Current smoking	23 (16.0)	8 (11.3)	0.356
Coronary artery occlusive disease	19 (13.2)	11 (15.5)	0.647
Atrial fibrillation	75 (52.1)	37 (52.1)	0.997
**Clinical conditions**			
Initial NIHSS score	14.0 [9.0; 19.0]	14.0 [8.5; 19.0]	0.756
Intravenous tPA administration	47 (32.6)	21 (29.6)	0.650
Location of occlusions			0.064
Internal carotid artery	42 (29.2)	30 (42.3)	
Middle cerebral artery	86 (59.7)	31 (43.7)	
Vertebral artery	3 (2.1)	0 (0.0)	
Basilar artery	13 (9.0)	10 (14.1)	
Onset-to-puncture time (minutes)	394.0 [180.0; 736.0]	311.0 [156.0; 636.0]	0.300
Use of balloon guide catheter	123 (85.4)	60 (84.5)	0.860
**Preprocedural D-dimer level (ng/mL)**	879.0 [437.0; 2748.0]	606.0 [268.0; 1062.0]	0.002
**Outcomes following first-pass effect**			
**Endovascular outcomes**			
Successful recanalization	132 (91.7)	71 (100.0)	0.010
Puncture-to-recanalization time (minutes)	40.5 [26.8; 66.2]	22.0 [14.0; 29.0]	<0.001
Complete recanalization	67 (46.5)	71 (100.0)	<0.001
Modified first-pass effect	19 (13.2)	71 (100.0)	<0.001
Thrombus fragmentation	79 (54.9)	0 (0.0)	<0.001
Number of passes of thrombectomy device	2.8 (±1.5)	1.0 (±0.0)	<0.001
**Favorable outcome**	49 (34.0)	38 (53.5)	0.006

Values represent the mean and standard deviation (±), median with the first and third quartiles [in brackets], or the number of patients (in %). NIHSS, National Institutes of Health Stroke Scale; tPA, tissue-type plasminogen activator.

**Table 4:** Comparison of clinical and endovascular findings according to favorable outcome.

	Favorable Outcome (−) (*n* = 128)	Favorable Outcome (+) (*n* = 87)	*p*-Value
**Demographics and stroke risk factors**			
Age (years)	73.5 (±12.3)	67.9 (±13.0)	0.002
Men	56 (43.8)	57 (65.5)	0.002
Hypertension	102 (79.7)	56 (64.4)	0.012
Diabetes	51 (39.8)	20 (23.0)	0.010
Dyslipidemia	23 (18.0)	31 (35.6)	0.003
Current smoking	17 (13.3)	14 (16.1)	0.565
Coronary artery occlusive disease	15 (11.7)	15 (17.2)	0.251
Atrial fibrillation	69 (53.9)	43 (49.4)	0.519
**Clinical conditions**			
Initial NIHSS score	16.0 [12.0; 20.0]	10.0 [6.0; 14.0]	<0.001
Intravenous tPA administration	33 (25.8)	35 (40.2)	0.025
Location of occlusions			0.712
Internal carotid artery	44 (34.4)	28 (32.2)	
Middle cerebral artery	66 (51.6)	51 (58.6)	
Vertebral artery	2 (1.6)	1 (1.2)	
Basilar artery	16 (12.5)	7 (8.1)	
Onset-to-puncture time (minutes)	426.0 [188.0; 793.0]	309.0 [166.0; 662.0]	0.106
Use of balloon guide catheter	105 (82.0)	78 (89.7)	0.123
**Endovascular outcomes**			
Successful recanalization	117 (91.4)	86 (98.9)	0.030
Puncture-to-recanalization time (minutes)	40.0 [22.0; 63.0]	28.0 [18.2; 37.8]	0.003
Complete recanalization	71 (55.5)	67 (77.0)	0.001
First-pass recanalization			
Modified first-pass effect	45 (35.2)	45 (51.7)	0.016
First-pass effect	33 (25.8)	38 (43.7)	0.006
Thrombus fragmentation	52 (40.6)	27 (31.0)	0.152
Number of thrombectomy device passes	2.4 (±1.6)	1.9 (±1.2)	0.006
**Preprocedural D-dimer level (ng/mL)**	1189.0 [526.0; 3208.0]	495.0 [290.0; 856.0]	<0.001

Values represent the mean and standard deviation (±), median with the first and third quartiles [in brackets], or the number of patients (in %). NIHSS, National Institutes of Health Stroke Scale; tPA, tissue-type plasminogen activator.

**Table 5:** Clinical and endovascular factors associated with favorable outcome.

	aOR (95% CI)	*p*-Value
Age	0.97 (0.94–0.99)	0.048 *
Men	2.31 (1.09–4.93)	0.030 *
Hypertension	0.60 (0.25–1.43)	0.246
Diabetes	0.34 (0.15–0.81)	0.014 *
Dyslipidemia	3.17 (1.34–7.48)	0.009 **
Initial NIHSS score	0.85 (0.79–0.91)	<0.001 ***
Intravenous tPA administration	2.28 (1.08–4.82)	0.031 *
Successful recanalization	16.3 (1.62–163.1)	0.018 *
First-pass effect	3.05 (1.17–7.94)	0.023 *
Number of thrombectomy device passes	1.14 (0.82–1.57)	0.440
Preprocedural D-dimer level (per 500 ng/mL)	0.88 (0.81–0.97)	0.008 **

* *p*-value < 0.05; ** *p*-value < 0.01; *** *p*-value < 0.001. aOR, adjusted odds ratio; CI, confidence interval; NIHSS, National Institutes of Health Stroke Scale; tPA, tissue-type plasminogen activator.

## Data Availability

The data presented in this study are available upon request from the corresponding author.
